# Bone marrow stromal cell antigen 2 (BST-2) restricts mouse mammary tumor virus (MMTV) replication *in vivo*

**DOI:** 10.1186/1742-4690-9-10

**Published:** 2012-01-27

**Authors:** Philip H Jones, Harshini V Mehta, Martina Maric, Richard J Roller, Chioma M Okeoma

**Affiliations:** 1 Department of Microbiology, University of Iowa, Carver College of Medicine, Iowa City, IA, USA

**Keywords:** BST-2, Tetherin, Interferon alpha, MMTV, *In vivo*, SEM, TEM

## Abstract

**Background:**

Bone marrow stromal cell antigen 2 (BST-2) is a cellular factor that restricts the egress of viruses such as human immunodeficiency virus (HIV-1) from the surface of infected cells, preventing infection of new cells. BST-2 is variably expressed in most cell types, and its expression is enhanced by cytokines such as type I interferon alpha (IFN-α). In this present study, we used the beta-retrovirus, mouse mammary tumor virus (MMTV) as a model to examine the role of mouse BST-2 in host infection *in vivo*.

**Results:**

By using RNA interference, we show that loss of BST-2 enhances MMTV replication in cultured mammary tumor cells and *in vivo*. In cultured cells, BST-2 inhibits virus accumulation in the culture medium, and co-localizes at the cell surface with virus structural proteins. Furthermore, both scanning electron micrograph (SEM) and transmission electron micrograph (TEM) show that MMTV accumulates on the surface of IFNα-stimulated cells.

**Conclusions:**

Our data provide evidence that BST-2 restricts MMTV release from naturally infected cells and that BST-2 is an antiviral factor *in vivo*.

## Background

Bone marrow stromal cell antigen 2 (BST-2) protein also known as tetherin/CD317 is a potent restriction factor against a wide range of enveloped viruses such as HIV, FIV, KSHV, MMTV, SIV, Lassa, Marbug, Ebola, and MLV [1-5]. BST-2 achieves its anti-viral effect by connecting both viral and host cell membranes, thus preventing virus egress [6-9]. While BST-2 inhibits virus release, most viruses including HIV-1, HIV-2, and Ebola virus have developed strategies to antagonize BST-2 by degradation, down-regulation of expression, or reduction of its steady-state level [1,2,7,10-14]. In addition to inhibiting virus egress and virus replication in cell culture, there is evidence that, following interferon-induction, BST-2 is up-regulated and incorporated into budding virions [2,6,8,9]. While the antiviral activity of BST-2 has been demonstrated in tissue culture cells, there has been no evidence that BST-2 exerts antiviral activity *in vivo*. In this context, we evaluated the ability of mouse BST-2 to restrict the replication of the exogenous murine retrovirus mouse mammary tumor virus (MMTV) in cell culture and in mice.

*In vivo*, MMTV first infects antigen presenting cells (APCs) such as B cells and dendritic cells (DCs) at the site of infection [15-19]. MMTV infected APCs present virus-encoded superantigen (Sag) to T cells expressing Sag-specific T-cell receptor (TCR) Vβ chains. This immunological synapse causes stimulation of Sag-reactive T cells and proliferation of lymphocytes thereby promoting virus replication. Both lymphoid and myeloid cells infected with MMTV are capable of producing infectious virus [19] and infected lymphoid cells are necessary for virus spread and mammary carcinogenesis [20,21].

Although MMTV infects and causes mammary cancer in infected mice; nonetheless, infected cells do not produce high virus titer, and time to MMTV-induced cancer is rather long, suggesting that virus replication and spread may be restricted by host factors like BST-2. Indeed, we demonstrate that BST-2 co-localizes with MMTV Gag and Env, and inhibits MMTV particle release in tissue culture cells. Importantly, MMTV infection of mice was significantly inhibited by BST-2.

## Results

### IFNα induces BST-2 expression and restricts MMTV release

**Figure 1:**
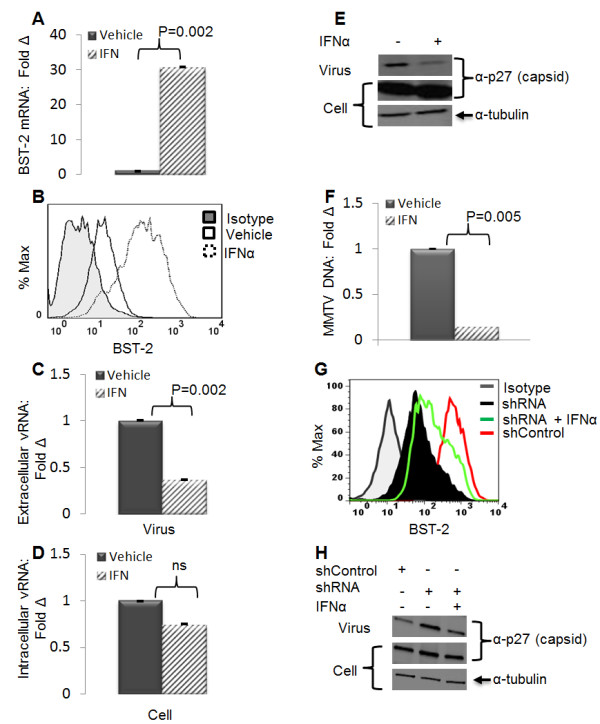
**IFNα-dependent induction of endogenous BST-2 restricts MMTV release**. GR cells were treated with 1000 units of endotoxin free recombinant IFNα or PBS for 24 hours. Virus was purified from culture supernatants and the corresponding cell extracts were harvested. (A) Cell extracts were used to determine level of BST-2 mRNA by qPCR, presented as fold change in BST-2 mRNA relative to vehicle treated cells; (B) and surface BST-2 by flow cytometry with the peak on the left as the isotype antibody control. Culture supernatants and their corresponding cell extracts were used to evaluate level of viral RNA, (C) extracellular viral RNA, (D) intracellular viral RNA, (E) and viral protein detected by antibody to MMTV capsid protein p27. Tubulin is a loading control. Data are presented as fold change in vRNA relative to vehicle treated samples. (F) Culture supernatants from IFNα or vehicle treated GR cells were used to infect TRH3 cells. Twenty four hours after infection, cells were harvested and used for DNA extraction followed by qPCR analysis of viral DNA normalized to GAPDH. Data are presented as fold change in viral DNA of cells infected with supernatants from IFNα treated cells relative to viral DNA of cells infected with supernatants from vehicle treated cells. Error bars are standard deviation, and p is the significance level, ns is not significant. Experiments were performed at least three times with similar results. (G) GR cells were stably transduced with shRNA targeting mouse BST-2 gene (shRNA) or a non-targeting shRNA (shControl). Cells were treated with IFNα (1000 units/ml) or vehicle. Twenty four hours later a portion of cells were used for FACS analysis of surface BST-2 level. (H) Culture supernatant and the remaining cells were examined for level of extracellular and intracellular viral proteins by Western blot.

BST-2 expression results in retention of a wide range of virus-like particles (VLPs) assembled in tissue culture [1-4], and IFNα or IFNγ treatment induces the transcription of the gene encoding BST-2 [1,22]. We hypothesized that IFNα induction of BST-2 in an MMTV producing cell line would enhance BST-2 expression and suppress MMTV release to the culture medium. We treated MMTV-producing GR cells with IFNα or vehicle and then measured BST-2 mRNA and surface protein levels, as well as extra- and intra- cellular viral particles. We observed a significant increase in BST-2 mRNA (Figure 1A) and protein (Figure 1B) levels upon IFNα treatment. This increase in BST-2 levels was accompanied by a reduction in the accumulation of MMTV particles in the culture supernatant, with no change in intracellular viral load as assessed by viral RNA (Figures 1C and 1D) and viral protein (Figure 1E). Additionally, the reduction in particle accumulation in culture supernatant results in lower viral load when such supernatants were used to infect MMTV susceptible cells (Figure 1F). This data demonstrate that IFNα induces endogenous BST-2 in MMTV infected cells and suggest that BST-2 may be the critical factor limiting the accumulation of MMTV particles in the culture supernatant.

Next, we examined whether BST-2 is responsible for IFNα-dependent inhibition of MMTV release. GR cells stably silenced for BST-2 expression with a lentiviral construct carrying BST-2 specific shRNA or control shRNA were stimulated with IFNα or vehicle. Culture supernatants were collected 24 hours later and subjected to Western blot analysis for MMTV capsid protein p27. The corresponding cells were examined for BST-2 protein (Figure 1G). Results show that while shRNA was able to silence BST-2 and enhance accumulation of viral particles in culture supernatant (Figure 1H), IFNα stimulation of shRNA cells results in increased cell surface BST-2 protein expression (Figure 1G) and a reduction in the amount of viral particles accumulated in the culture supernatant (Figure 1H). This result shows that the reduction in the amount of extracellular viral particles observed in IFNα treated cells is linked to BST-2 induction.

### IFNα causes accumulation of MMTV structures at the cell surface

**Figure 2:**
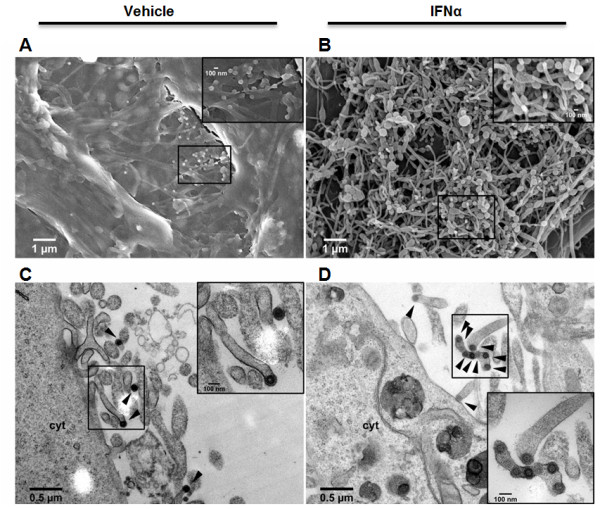
**Retention of MMTV virions on the cells surface of IFNα treated cells**. GR cells were treated with vehicle (left panels) or IFNα (right panels) for 24 hours and then processed for scanning (A and B) or transmission (C and D) electron microscopy. MMTV virions with a distinct capsid and fuzzy appearing envelope are indicated with black arrowheads (C and D). Each panel has indicated scale bar. Cyt is cytoplasm.

In IFNα treated cells, less MMTV particles were detected in the culture supernatant without apparent defect in accumulation of intracellular viral components (Figures 1E, 1H). As BST-2 was shown to block the release of various enveloped viruses/VLPs from the cells [1,23], we hypothesize that exposure of MMTV-producing GR cells to IFNα will result in accumulation of MMTV virions on the cell surface. To test this, GR cells were stimulated with IFNα or vehicle and 24 hours later, cells were processed for scanning electron microscopy (SEM). In vehicle treated GR cells, 100 nm sized buds were readily observed (Figure 2A). The buds were commonly found on the tip of filamentous structures projecting from the cells surface. IFNα induction drastically increased the number of bud-containing filaments (Figure 2B). To confirm that the buds were indeed virions, thin sections of GR cells prepared under the same treatment conditions were analyzed with transmission electron microscope (TEM). Electron dense virion structures, about 100 nm in diameter (black arrowheads), were detected at the cells surface (Figure 2C and 2D). As expected, in IFNα treated cells, particles that are linked to each other were also observed (Figure 2D). The TEM thus validated that the filamentous structures observed with SEM contain virion(s). Together the electron microscopy data strongly suggest that exposure of MMTV infected cells to IFNα results in retention of MMTV virions on the cell surface.

### BST-2 co-localizes with MMTV structural proteins at the cell surface

**Figure 3:**
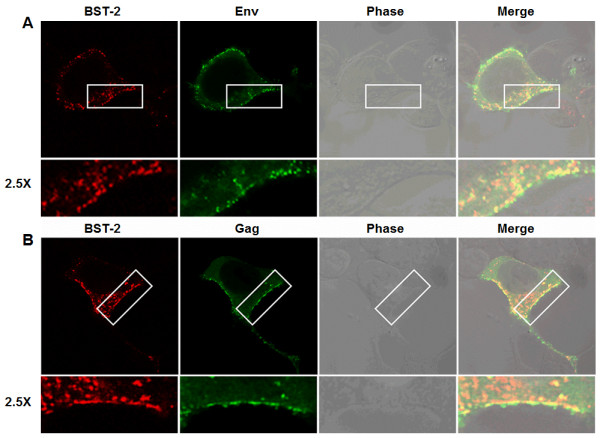
**MMTV Gag co-localizes with BST-2**. 293T cells transiently transfected with BST-2 and MMTV proviral constructs were permeabilized, fixed, stained, and analyzed with confocal microscopy for BST-2 and (A) MMTV Env or (B) MMTV Gag localization. Green is MMTV Env or Gag, red is BST-2, orange is merge. Phase contrast shows cell morphology. Areas of BST-2/Env or BST-2/Gag co-localization at the cells surface are highlighted in white rectangular boxes and magnified by 2.5X. Experiments were repeated at least three times with similar results.

Consistent with its role in tethering virus particles to the surface of the infected cell, BST-2 has been shown to co-localize with structural proteins of other retroviruses along the plasma membrane of cells [1,3]. To examine whether BST-2 co-localizes with MMTV structural proteins, BST-2, rat glucocorticoid receptor (RSVGR), and MMTV proviral genome (HP) were co-transfected into 293T cells. Two days later, cells were processed and immuno fluorescently stained to detect BST-2 and MMTV structural proteins. Results show partial co-localization of MMTV Env (Figure 3A) or Gag (Figure 3B) with BST-2 at the cell surface. Our data support previous demonstrations of co-localization of BST-2 with HIV-1 Env in infected cells [24] and co-localization of SIV Env and BST-2 in activated primary rhesus macaque lymphocytes infected with SIV [25]. In addition, our data are in line with previous report of BST-2 co-localization with the Gag proteins of other retro- and filo-viruses [3].

### Depletion of endogenous BST-2 enhances MMTV particle release and replication in cell culture

**Figure 4:**
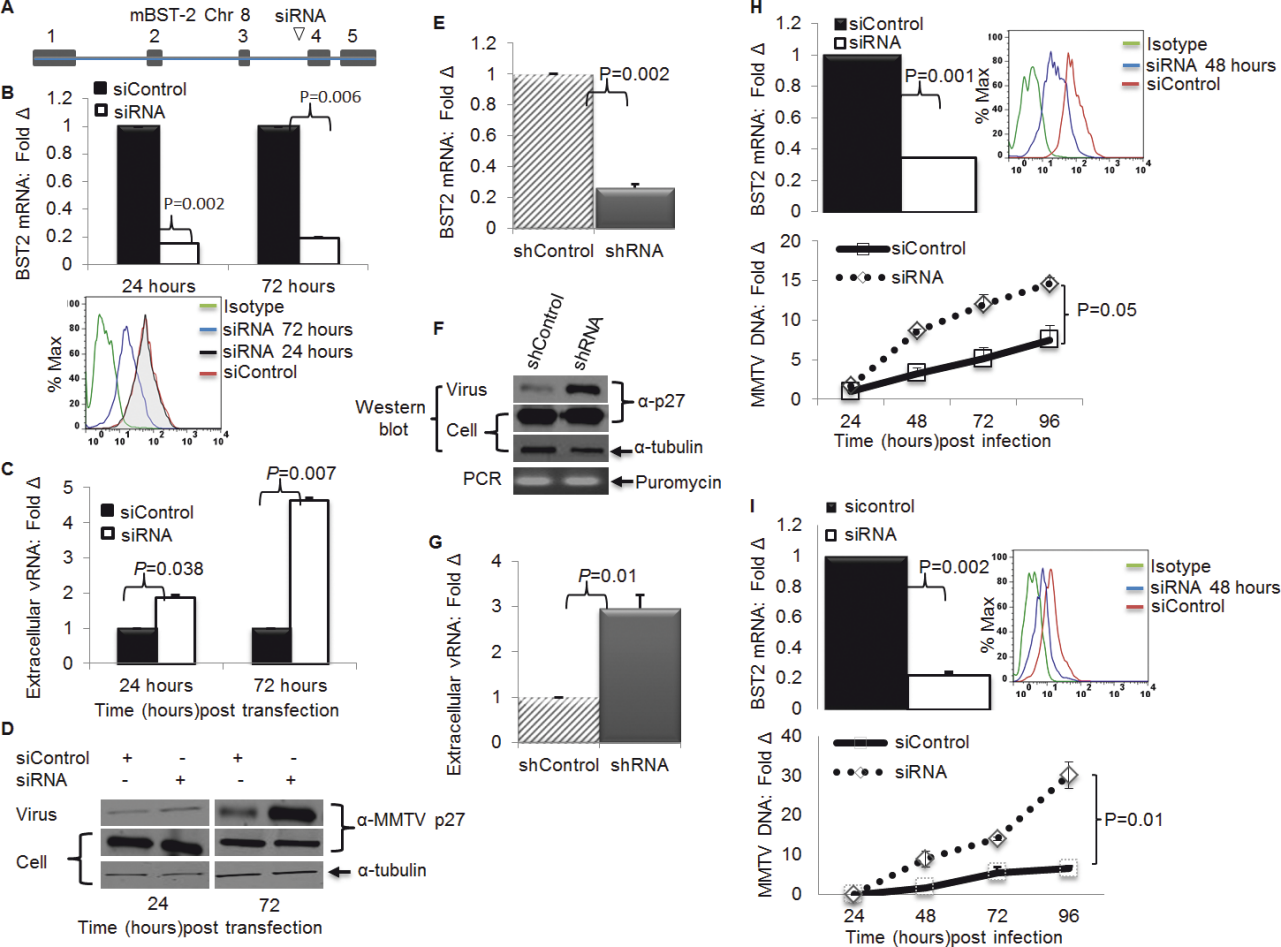
**Depletion of endogenous BST-2 enhances MMTV particle release and virus replication in tissue culture cells**. GR cells were transfected with siRNA targeting the mouse BST-2 gene or non-targeting control siRNA (siControl). At 24 or 72 hours after transfection, both culture supernatants and their corresponding cells were collected. Virions were purified from supernatants, and both virions and corresponding cells were used for RNA and protein extractions. Aliquots of the supernatants were used to quantify the amount of extracellular virus. (A) Location of the siRNA sequence on the BST-2 gene; (B) Level of BST-2 following siRNA treatment (upper panel, bars- BST-2 mRNA presented as fold change relative to the control and lower panel, histogram- BST-2 protein); (C) Level of extracellular viral RNA following treatment with BST-2 siRNA and siControl, presented as fold change relative to the control; (D) Western blot of extracellular and intracellular viral proteins detected by antibody to the capsid protein p27; tubulin is loading control. Next, GR cells were stably transduced with shRNA targeting mouse BST-2 mRNA or a non-targeting shRNA. (E) Cells were used to determine extent of BST-2 knockdown by qPCR. (F and G) Culture supernatants and their corresponding cells were used to examine level of (F) extracellular and intracellular viral proteins by Western blot, stable integration of lentiviral particles carrying shRNA was evaluated by puromycin PCR; (G) extracellular viral RNA. To evaluate the effect of BST-2 on MMTV replication, NMuMG cells or 3T3 MEF cells were transfected with siRNA or siControl. Twenty four hours after transfection, cells were infected with MMTV and harvested at different times (shown on the figures) for DNA extraction and qPCR analysis of viral DNA, (H) qPCR analysis of endogenous NMuMG BST-2 mRNA (top-left panel), cell surface expression (top-right panel), MMTV DNA (bottom-left panel); (I) qPCR analysis of endogenous 3T3 MEF BST-2 mRNA (top-left panel), cell surface expression (top-right panel), MMTV DNA (bottom-left panel) following knockdown with siRNA or siControl. Data are normalized to GAPDH and presented as fold change relative to siControl treated cells. Error bars are standard deviation, p is significance level. Experiments were repeated at least three different times with similar results.

To test the hypothesis that BST-2 is responsible for inhibition of MMTV release, we used an RNAi assay to determine whether silencing of endogenous BST-2 expression would lead to higher MMTV particle release and higher levels of infection. GR cells were transfected with either a control siRNA, or a siRNA that targets BST-2 mRNA (Figure 4A). Silencing endogenous BST-2 with siRNA (Figure 4B: upper panel - mRNA, lower panel - protein) enhanced extracellular viral RNA and protein yield compared to cells treated with control siRNA (Figure 4C and 4D respectively), suggesting that endogenous BST-2 affects MMTV particle release. Similar results were obtained when endogenous BST-2 was silenced in GR cells with lentivirus-based shRNA targeting mouse BST-2 (Figures 4E,F,G). Given the ability of endogenous BST-2 to regulate MMTV release, we wondered if BST-2 expression could affect replication and spread of MMTV. Hence, we depleted BST-2 with siRNA in mammary epithelial NMuMG cells (Figure 4H: left panel -mRNA, right panel - protein) and in 3T3 MEFs (Figure 4I: left panel - mRNA, right panel - protein). To evaluate the effect of reduced BST-2 levels on MMTV replication, we infected siRNA treated cells with MMTV, and observed significant differences in viral spread over a course of 96 hours in both NMuMG (Figure 4H: bottom panel) and 3T3 MEFs (Figure 4I: bottom panel). These data suggest that BST-2 impairs MMTV replication and spread in epithelial and fibroblast cells.

### Overexpression of BST-2 inhibits MMTV replication

**Figure 5:**
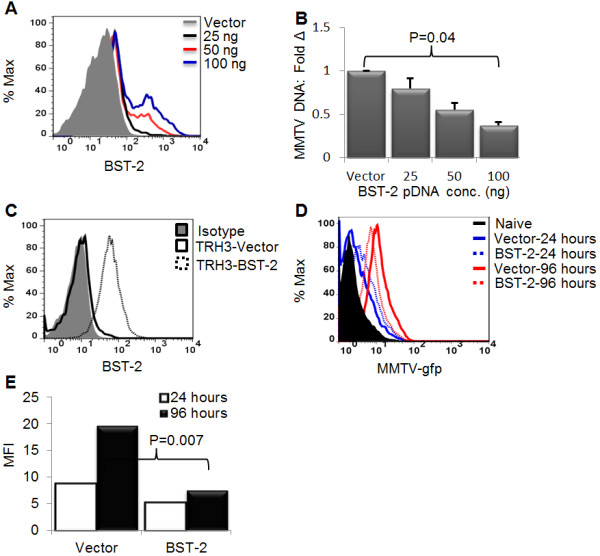
**Ectopic expression of BST-2 inhibits MMTV replication in tissue culture cells**. (A-B) Varying concentrations of BST-2 were transiently transfected into TRH3 cells. Twenty four hours later, (A) a portion of cells were processed for FACS analysis and evaluated for BST-2 surface expression and (B) the remaining portion was infected with MMTV and examined for level of infection by qPCR 24 hours after infection. Data are normalized to GAPDH and presented as fold change in viral DNA relative to viral DNA of vector transfected cells. (C-E) Constructs of BST-2 and puromycin were stably co-expressed in TRH3 cells. Twenty four hours later, fresh medium containing puromycin drug was added to cells to facilitate the selection of transfectants. After about 10 days in selection, individual colonies of transfected cells were picked and amplified. A portion of cells were examined for (C) BST-2 surface expression by FACS. Another portion of cells were infected with MMTV-gfp. Twenty four and ninety six hours after infection, cells were analyzed for infection as determined by (D) level of gfp expression or (E) intensity of gfp expression using FACS. Error bars are standard deviation, p is significance level. All experiments were repeated at least three different times with similar results.

To confirm our finding that BST-2 restricts MMTV replication in cultured cells, we transiently expressed varying amounts of BST-2 or empty vector in TRH3 (293T cells that stably express MMTV entry receptor transferrin receptor 1). Twenty four hours later, a portion of cells were examined for BST-2 expression (Figure 5A), and the remaining cells were infected with MMTV. DNA was extracted from infected cells and used for qPCR examination of virus load 24 hours after infection. Results show that while cells transfected with BST-2 plasmid show lower levels of MMTV DNA across board, transfection of 100 ng of BST-2 plasmid results in about 60% lower infection (Figure 5B), indicating that BST-2 restricts MMTV replication in a dose dependent manner. Whether lower infection observed in BST-2 transfected cells is a result of cells being infected at low multiplicity or lower number of cells being infected is unclear. To address this question, we first generated TRH3 cells stably expressing BST-2 or empty vector (Figure 5C) and infected cells with MMTV.*gfp *purified from MMTV-producer cell line, CGRES6. These cells produce virions derived from a molecular clone of MMTV (pGR102ES) with a gfp gene inserted into its long terminal repeat (LTR) [26]. Infected cells were collected 24 and 96 hours later and examined for level and intensity of gfp expression. Results show that both at 24 and 96 hours after infection, level of gfp expression was significantly lower in BST-2 expressing cells compared to cells expressing empty vector control (Figure 5D). In addition, gfp mean fluorescence intensity (MFI) was also lower in cells expressing BST-2 at both time points (Figure 5E). These results suggest that BST-2 potently restricts MMTV replication as evidenced by the larger proportion of cells infected in the absence of BST-2 (Figure 5D, solid blue line and solid red line) compared to lower number of cells infected in the presence of BST-2 (Figure 5D, broken blue line and broken red line).

### BST-2 specific siRNA depleted endogenous BST-2 in murine lymph nodes

**Figure 6:**
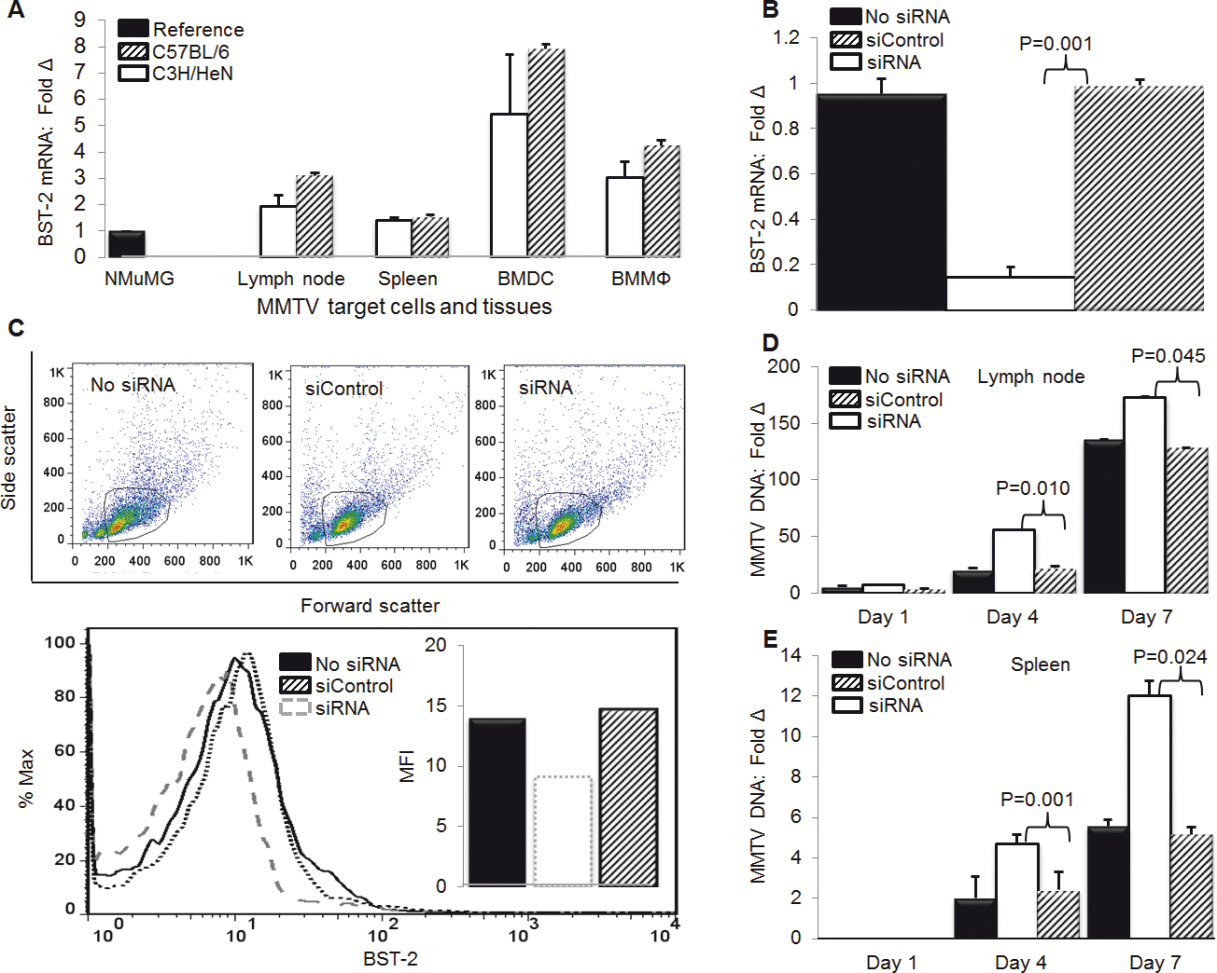
***In vivo *replication of MMTV is enhanced in the absence of BST-2**. (A) Naïve female age-matched C3H/HeN (n = 3) and C57BL/6 (n = 3) were profiled for BST-2 expression by qPCR analysis of BST-2 mRNA levels in popliteal lymph node, spleen, bone marrow derived DCs (BMDC), and bone marrow derived macrophages (BMMΦ). Data are presented as fold change in BST-2 level relative to NMuMG BST-2. (B-E) Naïve age-matched C3H/HeN mice were inoculated with siRNA (n = 5), siControl (n = 5), or PBS (No siRNA, (n = 5) subcutaneously on the hind foot pad. Forty-eight hours after inoculation, mice were infected with MMTV on the same footpad. Cells of the draining popliteal lymph nodes (B-D) or spleen (E) were used for DNA and total RNA extraction or flow cytometry. (B) qPCR analysis of BST-2 mRNA 48 hours after siRNA inoculation, presented as fold change in BST-2 level relative to siControl treated mice (C) Flow cytometric analysis of surface BST-2 following *in vivo *siRNA treatment showing scatter plot of the gated cell population (upper panels) and % of BST-2 expressing cells (lower panel). Inset in the lower panel is mean florescence intensity - MFI. (D) qPCR analysis of proviral DNA in the popliteal lymph node of infected mice, presented as fold change in viral DNA relative to viral DNA in siControl lymph node at 24 hours after infection. (E) qPCR analysis of proviral DNA in the spleen of infected mice, presented as fold change in viral DNA relative to viral DNA in siControl spleen at 24 hours after infection. Error bars are standard deviation, p is significance level. All experiments were performed with either 3 or 5 mice per group and repeated at least three times with similar results.

We have demonstrated that loss of BST-2 in tissue culture cells enhances MMTV replication and spread (Figures 4H and 4I lower panels). To examine the role of BST-2 in virus infection *in vivo*, we first determined whether MMTV target cells and tissues such as dendritic cells (DCs), macrophages (MΦs), lymph nodes and spleens from different mouse strains (C3H/HeN and C57BL/6) that express BST-2 mRNA. We used the mammary epithelial cell line, NMuMG as a reference and show that BST-2 transcript is present in all MMTV targets (Figure 6A). BST-2 mRNA is higher in bone marrow derived DC (BMDC) compared to bone marrow derived macrophages (BMMΦ), popliteal lymph nodes and the spleen, with a modest difference in BST-2 levels between C3H/HeN and C57BL/6 mice. Since lymph nodes express BST-2, we examined whether BST-2 specific siRNA is capable of silencing BST-2 *in vivo*. Following inoculation of mice with siRNA or siControl subcutaneously in the hind footpad, we evaluated the levels of BST-2 mRNA and protein and show that in 48 hours, siRNA significantly depleted lymph node BST-2 mRNA to about 7 fold (Figure 6B) with a modest reduction in protein level (Figure 6C: upper and lower), and no effect on BST-2 levels of mice inoculated with siControl or no siRNA. We also examined the expression of IFNα, IFNβ, and 2'-5'-oligoadenylate synthetase (OAS) transcripts, and observed that mice treated with siRNA sequences have similar levels of IFNα, IFNβ, and OAS transcripts despite reduced BST-2 mRNA level (Additional file 1). These data suggest that BST-2 specific siRNA reduced BST-2 expression *in vivo *without eliciting IFN response.

### siRNA mediated silencing of endogenous BST-2 enhances MMTV replication and spread *in vivo*

Local administration of BST-2 siRNA mediates BST-2 depletion *in vivo *(Figures 6B and 6C). To determine the role of endogenous BST-2 in virus replication *in vivo*, we administered either a control siRNA, or siRNA that targets BST-2 mRNA to mice, followed by infection with MMTV. Virus replication was significantly higher in siRNA treated mice compared to their siControl or no siRNA counterparts (Figure 6D). In addition, we observed significantly higher virus load in the spleens of mice that received siRNA (Figure 6E). These data show for the first time that siRNA is capable of mediating BST-2 knock down *in vivo *(Figures 6B and 6C); and that loss of BST-2 in the draining lymph node for the site of initial infection results in higher rate of virus replication and dissemination *in vivo *(Figures 6D and 6E).

## Discussion

Here, we describe an important anti-viral function for BST-2 in the mouse. Many *in vitro *and *ex vivo *studies have shown that BST-2 inhibits the replication of a number of viruses [1-9]. By using MMTV, we directly demonstrate that BST-2 plays a role in virus replication *in vivo*. Administration of siRNA sequences into mouse footpad results in knock-down of endogenous lymph node BST-2. This assay allowed us to demonstrate that BST-2 functions as a virus restriction factor in a natural host organism. We chose to silence lymph node BST-2 because lymphocytes play a critical role in the *in vivo *infection with MMTV and other retroviruses such as HIV-1; and in the absence of a knockout model, siRNA provides the best alternative. The significantly higher virus load observed in BST-2 silenced lymph nodes implies that loss of BST-2 enhances virus replication.

BST-2 expression inhibits the egress of HIV-1, HIV-2, Ebola, Marburg and MLV; and we found that it also inhibits the accumulation of MMTV particles in the culture medium. The inhibitory effect of BST-2 on accumulation of extracellular MMTV particles in culture supernatant is consistent with the effect of BST-2 on virus release from the cell surface as has been observed for other viruses [1-5]. The observation that BST-2 co-localizes with MMTV Env and Gag further suggests that BST-2 prevents the accumulation of MMTV particles in culture supernatants. Our EM data support previously documented studies that MMTV buds off from the plasma membrane and microvilli [27]. The cell surface visualization by SEM provides an opportunity to demonstrate that induction of BST-2 with IFNα facilitates accumulation of MMTV on the cell surface. This finding was backed by our protein analyses showing that stimulation of MMTV producer cells with IFNα results in less accumulation of MMTV particles in the culture supernatant.

## Conclusion

We have directly demonstrated in this study that loss of BST-2 enhances replication of MMTV *in vivo*. However, it remains to be determined why mice become infected with MMTV in the presence of the endogenous BST-2. It is worth noting that MMTV has co-existed with mice for over 20 million years [28] and perhaps must have evolved to avoid most hosts' anti-viral defense mechanisms. The findings of this study are important as it has shown that BST-2 function as anti-viral in the host. Based on this report, we are now focused on using MMTV as a model to understand the role of BST-2 in viral pathogenesis.

## Materials and methods

### Ethics statement

All experiments involving mice were performed in accordance to NIH guidelines, the Animal Welfare Act, and US federal law. The experiments approved by the University of Iowa Animal Care and Use Committee (IACUC). Mice were housed according to the policies of the Institutional Animal Care and Use Committee of the University of Iowa.

### Molecular constructs

BST-2 plasmids used in the study were previously described [4] and kindly provided by Dr. Paul Bates, University of Pennsylvania, Philadelphia, PA. The MMTV *env *(Q61) construct [20], MMTV molecular clone HYB PRO (HP) [29], and the rat glucocorticoid receptor (RSVGR) [30] were kindly provided by Dr. Susan Ross, University of Pennsylvania, Philadelphia, PA.

### Animals

C57BL/6 and C3H/HeN mice were purchased from the Jackson Laboratory and National Cancer Institute (NCI) respectively.

### Cell lines

NMuMG (derived from normal mammary tissue of a Namru mouse strain), GR (a mammary carcinoma cell line), Mm5MT (a mammary carcinoma cell line from C3H/HeN mouse strain), 3T3 MEF and 293T cell lines were purchased from American Type Culture Collection (ATCC). TRH3 cells are 293T-mTfR1 (293T cells that stably express MMTV entry receptor transferrin receptor 1 [mTfr1]) and have been previously described [31]. TRH3-BST-2 and TRH3-Vector cells (TRH3 cells stably expressing BST-2 or empty vector). CGRES6-GFP cells (feline kidney stably transfected with a GFP-tagged MMTV proviral construct [26] was obtained from Dr. Susan Ross of the university of Pennsylvania. All cells were maintained according to the suppliers' instructions. Both GR and Mm5MT cells are from MMTV-induced mammary tumor and both produce infectious MMTV particles.

### Generation of TRH3 cell-line stably expressing mBST-2

To generate a TRH3-BST2 stable cell line, we co-transfected 6 μg of BST-2 and 1 μg of puromycin (selection marker) plasmids into TRH3 cells using polyethylenimine (PEI) transfection reagent. Following overnight incubation, transfection medium was changed, and replaced with medium containing puromycin dihydrochloride (3 μg/ml, Santa Cruz Biotechnology, Inc.). The cells were left in culture with three changes of medium to remove dead cells. By day 10 after transfection, several individual clones of stably transfected cells emerged and clones were picked and expanded. Level of BST-2 expression was examined by FACs analysis. After examination, clones with the highest BST-2 expression were expanded and some cells frozen or used for experiments.

### Antibodies, siRNAs, shRNAs, and other reagents

Antibodies used in this study includes goat anti-gp52 (Env) and anti-p27 (capsid) previously described [32,33]. Others are unconjugated rat anti-mouse BST-2 clone 129c, Alexa flour 647 conjugated anti-mouse BST-2 clone 129c [34], and FITC conjugated anti-mouse BST-2 clone 927 purchased from eBioscience. Rabbit anti-BST-2 and mouse anti-tubulin were from Abnova and Li-core respectively. The following isotype control antibodies were used: Rat IgG2a and Rat IgG2b, both from eBiosciences. The following secondary antibodies from Li-core were used: RDye 680 donkey anti-mouse, IRDye 680 LT conjugated donkey anti-rabbit, IRDye 800CW donkey anti-goat, and IRDye 800CW conjugated goat anti-rabbit. Rabbit anti-rat Alexa Fluor 594 and chicken anti-goat Alexa Fluor 488 were from Invitrogen. The *in vivo *ready silencer Select target specific siRNA, control siRNA, and SiPort NeoFX transfection reagent were from Ambion while plasmids for target-specific Mission shRNA, control shRNA, and polybrene were obtained from Sigma. BST-2 shRNA and control shRNA lentiviral particles were produced from plasmids of target-specific Mission shRNA and control shRNA by the University of Iowa Vector Core Facility. Recombinant mouse interferon alpha (IFN-α) was from Miltenyi Biotech. Vector-shield was from Vector Laboratories and Lipofectamine 2000 was from Invitrogen.

### Infection of mice

*In vivo *infection of mice was performed with 50 μl of MMTV.MM5MT inoculated subcutaneously on the hind footpad. At different times after infection, mice were sacrificed and the popliteal lymph node draining the site of infection from each mouse was harvested. Lymphocytes were obtained and used for fluorescence-activated cell-sorting (FACS) analysis or used for DNA and RNA extraction.

### BST-2 knockdown by shRNA and siRNA in cell culture

BST-2 shRNA lentiviral particles containing 3 target-specific constructs and shRNA constructs encoding a scrambled sequence (Santa Cruz Biotechnology, Santa Cruz, CA) or target specific Mission shRNA and a corresponding control shRNA (Sigma) were used to knock down BST-2 gene expression. Briefly, mouse mammary epithelial tumor cell line GR were transduced with control or BST-2 shRNA lentiviral particles at a ratio of 5 infectious units of virus per cell in the presence of 8 μg/mL polybrene. The next day, fresh media was added to cells and incubated for another 24 hours. In some experiments, puromycin at 2 μg/ml was added to the culture medium for the selection of cells that had stably incorporated shRNA. BST-2 gene silencing was confirmed at the mRNA and protein levels by qPCR and FACS. Knockdown of BST-2 by siRNA was achieved using Ambion's *in vivo *ready silencer Select target specific or siRNA *in vivo *ready scrambled siRNA. Briefly, 250 pico moles of siRNA sequences were mixed with Lipofectamine 2000 following manufacturer's recommendation and used to transfect NMuMG, GR or MEFs. The next day, fresh medium was added to cells and incubated until assayed for BST-2 and MMTV mRNA. NMuMG and MEFs were passaged and used for detection of BST-2 and virus replication experiments. Experiments were repeated at least 3 times with similar results.

### Western blots

Western blots of virus preparations, cell lysates from MMTV infected cells, cells transiently transfected with *env *and capsid plasmids, or immuno-precipitates of protein complexes were probed with anti-total MMTV, anti-gp52, and anti-p27. The species-appropriate IRDye secondary antibodies were used, followed by detection with the Odyssey Infrared Imaging System (LI-COR Biosciences).

### DNA, RNA isolation and real-time quantitative PCR (qPCR)

DNA or total RNA was isolated from cells, tissues, and mice lymphocytes using the QIAGEN-QIAamp DNA Mini Kit or RNeasy Mini Kit (Qiagen, Inc.) respectively according to manufacturer's instructions. For cDNA synthesis, equivalent amounts of RNA treated with DNase I (Qiagen, Inc.) were reverse-transcribed with high capacity cDNA reverse transcription Kit (Applied Biosystems, ABI), and the cDNA was amplified with primers specific to BST-2, MMTV.Mm5MT, IFNα, IFNβ, 2'-5'-OAS, and GAPDH. DNA was used to quantitatively detect integrated proviruses [32,33], using primers specific to the MMTV long terminal repeats and puromycin. Semi-quantitative PCR was performed using Veriti 96-Well Thermal Cycler, and real time qPCR was carried out using ABI 7500 FAST thermal cycler (ABI). All amplifications were normalized to GAPDH and data are presented as fold change relative WT or naïve sample depending on experiment. Sequence of primer pairs used is available (Table 1).

**Table 1:** Primer sequences

Primer name	Forward sequence	Reverse sequence
mBST-2	TCAGGAGTCCCTGGAGAAGA	ATGGAGCTGCCAGAGTTCAC

MMTV	CGTGAAAGACTCGCCAGAGCTA	TAATGTTCTATTAGTCCAGCCACTGT [32,33]^*a*^

GAPDH	CCCCTTCATTGACCTCAACTACA	CGCTCCTGGAGGATGGTGAT [32,33]^*a*^

Puromycin	CGTACGCACCCTCGCCGC	TCGTCCGCGACCCACACC

IFNα	GCTAGGCTCTGTGCTTTCCTGATG	CTCAGGTACACAGTGATCCTGTGG

IFNβ	AACAGGTGGATCCTCCACGCTGCG	GTGGAGAGCAGTTGAGGACATCTCC

2'-5'-OAS	CTTTGATGTCCTGGGTCATGT	CTCCGTGAAGCAGGTAGAG

Sequence of primer pairs used in this study. Citation means the primer has been previously reported.

### Immuno-fluorescence and confocal microscopy

293T cells were co-transfected with plasmids expressing BST-2, rat glucocorticoid receptor (RSVGR) and MMTV proviral genome (HP) using lipofectamine 2000. The next day cells were treated with DEX and 24 hours later fixed in 4% paraformaldehyde in PBS for 10 minutes. Cells were then washed once with PBS and incubated for 3 hours in IF buffer (0.5% Triton X-100, 0.5%, sodium deoxycholate, 1% bovine serum albumin, 0.05% sodium azide in PSB). Primary antibodies were diluted in IF buffer as follows: rat anti-BST-2 (1:100), goat anti-p27 (1:100) or goat anti-gp52 (1:100) and detected with rabbit anti-rat Alexa Fluor 594 and chicken anti-goat Alexa Fluor 488. All secondary autobodies were dilated 1:1000 in IF buffer. All confocal microscopy images were taken with Zeiss 510 confocal microscope.

### Flow cytometry

Approximately, 1 × 10^6 ^GR, MM5MT, NMuMG, 3T3 MEF, 293T, and lymphocytes were stained in PBS + 1% bovine serum albumin (Sigma-Aldrich) for 30 min on ice with Alexa flour 647 anti-mouse BST-2 (clone 129c) or FITC anti-mouse BST-2 (clone 927) and APC anti-mouse IgG2b. Cells were extensively washed in PBS, fixed with 2% paraformaldehyde and subjected to FACS. At least ten-thousand events were collected for each sample using FACS calibur flow cytometer (BD). Cellular frequency and mean fluorescence intensity (MFI) were determined by Flowjo analysis software (TreeStar).

### Virus release assay

GR cells were induced for virus production with 1 μM of dexamethasone (DEX) [35] and treated with IFNα or vehicle for 24 hours. Culture supernatants from cells were clarified by low speed centrifugation, passed through a 0.45 μm filter, and virions were pelleted through a 20% sucrose layer at 32,000*g *as previously described (30-31). Pellets of virus particles and corresponding cell extracts were denatured and analyzed by SDS-PAGE and Western blot assays. In some experiments, virus particles were used for qPCR analysis of viral RNA or to infect TRH3 cells.

### Statistical analysis

Statistical analysis of significant differences between experimental groups was tested using paired two-tailed Student's t test, and a p value of 0.05 was considered significant. Error bars represent standard deviations.

## List of abbreviations

BST-2: bone marrow stromal cell antigen 2; HIV-1: human immunodeficiency virus type 1; HIV-2: human immunodeficiency virus type 2; IFNα: interferon alpha and beta, siRNA: short interfering RNA; shRNA: short hair pin RNA; OAS: oligoadenylate synthetase; NMuMG: normal murine mammary gland; SIV: simian immunodeficiency virus; SEM: scanning electron micrograph; TEM: transmission electron micrograph.

## Competing interests

The authors declare that they have no competing interests.

## Authors' contributions

CMO conceptualized and designed research, PHJ, HVM, MM, CMO performed research, and analyzed data; PHJ, HVM, MM, RJR, CMO wrote and read the paper, RJR, Contributed reagents. All authors reviewed the manuscript and approved the final version.

## Authors' information

*Department of Microbiology, Carver College of Medicine, University of Iowa, Iowa City, IA 52242, United States of America.

## Supplementary Material

Additional file 1 **BST-2 siRNA did not elicit interferon response in mice**. Age-matched C3H/HeN mice were inoculated with siRNA (n = 3), siControl (n = 3), or PBS (WT, (n = 3) subcutaneously on the hind foot pad. Forty-eight hours after inoculation, mice were sacrificed and cells of the draining popliteal lymph node used for total RNA extraction. Quantitative PCR was used to examine mRNA levels of (A) BST-2 (B) IFNα, (C) IFNβ, and (D) 2'-5'-oligoadenylate synthetase (OAS). Data is presented as fold change relative to WT mice. Error bars are standard deviation, and p is significance level. Experiments were performed with 3 mice per group and repeated at least three times with similar results. Click here for file
