# Supplementary figures and images for: Bone marrow stromal cell antigen 2 (BST-2) restricts mouse mammary tumor virus (MMTV) replication in vivo
Source: Retrovirology. 2012 Jan 27;9:10. doi: 10.1186/1742-4690-9-10 (PMC3283513; doi:10.1186/1742-4690-9-10)

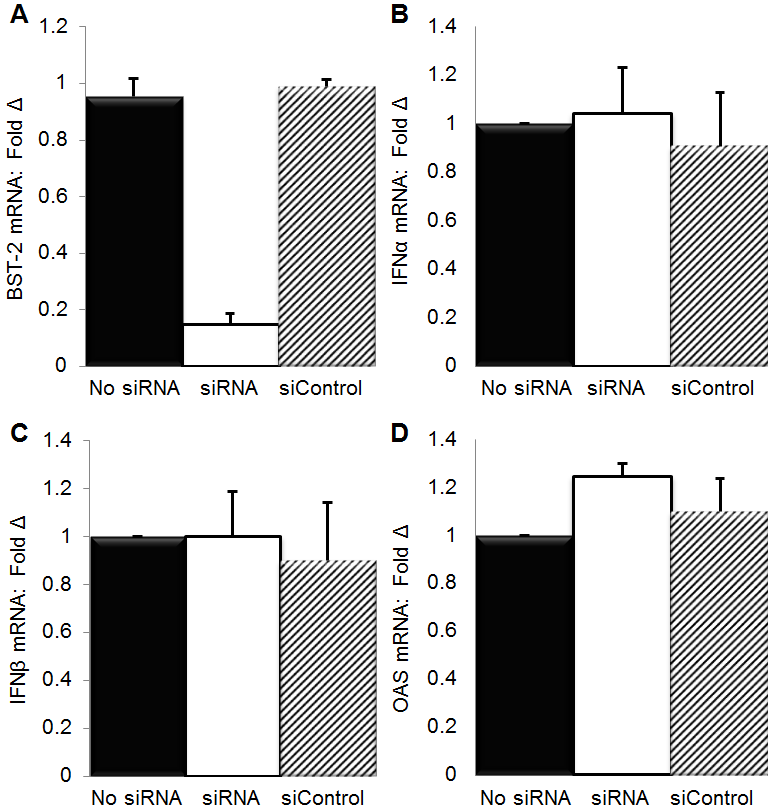

Supplement: Additional file 1 — BST-2 siRNA did not elicit interferon response in mice. Age-matched C3H/HeN mice were inoculated with siRNA (n = 3), siControl (n = 3), or PBS (WT, (n = 3) subcutaneously on the hind foot pad. Forty-eight hours after inoculation, mice were sacrificed and cells of the draining popliteal lymph node used for total RNA extraction. Quantitative PCR was used to examine mRNA levels of (A) BST-2 (B) IFNα, (C) IFNβ, and (D) 2'-5'-oligoadenylate synthetase (OAS). Data is presented as fold change relative to WT mice. Error bars are standard deviation, and p is significance level. Experiments were performed with 3 mice per group and repeated at least three times with similar results. [file 1742-4690-9-10-S1.TIFF]
